# Effects of Sourdough on FODMAPs in Bread and Potential Outcomes on Irritable Bowel Syndrome Patients and Healthy Subjects

**DOI:** 10.3389/fmicb.2018.01972

**Published:** 2018-08-21

**Authors:** Leidiane A. A. Menezes, Fabio Minervini, Pasquale Filannino, Maria L. S. Sardaro, Monica Gatti, Juliano De Dea Lindner

**Affiliations:** ^1^Department of Food Science and Technology, Federal University of Santa Catarina, Florianópolis, Brazil; ^2^Department of Soil, Plant and Food Sciences, University of Bari Aldo Moro, Bari, Italy; ^3^Department of Human Science and Promotion of the Quality of Life, University of San Raffaele, Rome, Italy; ^4^Department of Food and Drug, University of Parma, Parma, Italy

**Keywords:** fermentable oligosaccharides, disaccharides, monosaccharides and polyols, irritable bowel syndrome, bread, lactic acid bacteria, yeasts, sourdough

## Abstract

**Background:** Fermentable oligosaccharides, disaccharides, monosaccharides and polyols (FODMAPs) are an heterogeneous group of compounds that can be poorly digested and may have a range of effects on gastrointestinal processes. FODMAPs are found in a wide variety of foods, including bread. FODMAPs’ intake is associated with the onset of symptoms of irritable bowel syndrome (IBS). On the other hand, some FODMAPs contribute to the healthy maintenance of intestinal microbiota. Volume increase of bread dough commonly relies on the use of two biological leavening agents, sourdough and baker’s yeast and, in some cases, a combination of both.

**Scope and Approach:** The main objective of this review is to discuss the association between FODMAPs and IBS, beneficial effects of FODMAPs on healthy subjects and potential impact of biological leavening agents on FODMAPs content of bread.

**Key Findings and Conclusion:** Given that yeasts and lactic acid bacteria, the dominant microorganisms in sourdough, may degrade FODMAPs, it would be possible to modulate the FODMAPs concentration in bread, thus positively affecting consumers’ health.

## Introduction

Nowadays, tailored nutritional recommendations may be designed in order to treat or prevent diseases (Betts and Gonzalez, 2016). For instance, lactose- and gluten-containing foods must be avoided by subjects suffering from hypolactasia and celiac disease (De Toro-Martín et al., 2017). Fermentable oligosaccharides, disaccharides, monosaccharides and polyols (FODMAPs) are an heterogeneous group of compounds (most of which are short-chain carbohydrates) that can be poorly digested and may have a range of effects on gastrointestinal processes. This group includes lactose, fructose in excess of glucose, fructans, and fructooligosaccharides (FOS, such as nystose and kestose), galacto-oligosaccharides (GOS such as raffinose and stachyose), and sugar polyols (sorbitol and mannitol) (Muir et al., 2009).

Fermentable oligosaccharides, disaccharides, monosaccharides and polyols are found in a wide variety of foods. Their dietary uptake mostly results from honey and fruits as watermelons, pears, and apples (fructose); milk and dairy products (lactose); rye, wheat, artichoke, garlic, onions, and broccoli (fructans and FOS); pulses (GOS); stone fruits and artificial sweeteners, mushrooms, broccoli, and cauliflower (sugar polyols) (Muir et al., 2009; Shepherd et al., 2013).

Fructans and FOS are the main FODMAPs in wheat-based products (Biesiekierski et al., 2011; Shewry and Hey, 2015; Ziegler et al., 2016). They are composed of fructose molecules linked to each other through fructosyl–fructose bonds and of one sucrose terminal end. Inulin-type and levan-type are linear fructans containing β-(1/2) and β-(6/2), respectively, fructosyl–fructose bonds. Both bonds are found in branched chain fructans called “graminans” (Verspreet et al., 2015). These bonds may be cleaved by several enzymes: endo-inulinase, exo-β-fructosidase, endo-levanase, β-fructofuranosidase (alias invertase), and levansucrase. Endo-inulinase and endo-levanase act on internal linkages, generating oligosaccharides with a lower degree of polymerization. Exo-β-fructosidase and β-fructofuranosidase liberate terminal fructose. Invertase, typically splitting sucrose into glucose and fructose, could act on fructans and FOS (Verspreet et al., 2013). Levansucrase generates sucrose from FOS and could further split sucrose into glucose and fructose (De Angelis et al., 2017). GOS are soluble, non-reducing α(1,6) galactosyl extensions of sucrose. From a chemical point of view, it would be more correct to name these FODMAPs as “Raffinose Family of Oligosaccharides,” but they are usually referred to as “GOS.” Elongation of the trisaccharide raffinose with galactose residues leads to stachyose and verbascose (Van Den Ende, 2013). α-galactosidase acts on the α-1,6 linkage, thus hydrolyzing GOS (Katrolia et al., 2014). In addition, inulinase, invertase, and levansucrase may cleave the bond between fructose and glucose in sucrose, raffinose, stachyose, and verbascose to yield glucose, melibiose, manninotriose, and manninotetraose, respectively. Thus, the combined action of α-galactosidase and enzymes cleaving the fructose-glucose bond converts non-digestible GOS to carbohydrates with lower polymerization degree and potential prebiotic activity (Teixeira et al., 2012; Morel et al., 2015).

Fermentable oligosaccharides, disaccharides, monosaccharides and polyols’ intake may be detrimental for health, being associated, for instance, with the onset of symptoms of irritable bowel syndrome (IBS) (Barrett, 2017). On the other hand, some FODMAPs are proven to be prebiotic, contributing to the healthy maintenance of intestinal microbiota (Muir et al., 2009; Halmos et al., 2014). Wheat-based products (e.g., bread, breakfast cereals, and pasta) account for a major part of daily consumed FODMAPs, due to their high consumption as staple foods worldwide (Verspreet et al., 2015). In particular, since ages of ancient civilizations (Babylonians, Egyptians, Greeks, and Romans), bread has been a major component of daily diet of several peoples (Chavan and Chavan, 2011). Volume increase of dough (flour and water) usually occurring during bread-making commonly relies on the use of biological leavening agents. Sourdough is the traditional leavening agent, resulting from the fermentation of cereal flour and water, by mainly lactic acid bacteria (LAB) and yeasts (De Vuyst et al., 2014; Gobbetti et al., 2014; Minervini et al., 2014).

Starting from the beginning of the twentieth century, sourdough was gradually replaced by baker’s yeast (consisting of cell biomass mainly belonging to the yeast species *Saccharomyces cerevisiae*), a microbial starter culture produced at industrial level and distributed to bakers, which add it at low percentages (0.5–2.5%) in bread dough to obtain leavening. The main consequence of this revolution was a decreased flavor, due to reduced fermentation times and almost exclusive yeast metabolic activities (Cauvain and Young, 2009). It has also been hypothesized that short fermentation may have contributed to bread intolerance through its effects on fermentation in the colon (Costabile et al., 2014). However, during the two last decades, an increasing number of consumers is demanding traditional bread endowed with higher aroma and taste and the main industrial reply consists in the rediscovery of sourdough fermentation (Decock and Cappelle, 2005; Cauvain and Young, 2009). This review focuses on the association between FODMAPs and IBS, beneficial effects of FODMAPs on healthy subjects and potential impact of biological leavening agents on FODMAPs content of bread.

## IBS, FODMAPs, and Diet

IBS is one of the most common type of functional bowel disorders, in which abdominal pain is associated with a change in bowel habits (Giorgio et al., 2015). Symptoms include diarrhea, constipation, bloating, distension, abdominal discomfort and/or pain and flatulence (Tuck et al., 2014; Marsh et al., 2016). The prevalence of IBS is estimated between 7 and 21% worldwide (Brandt et al., 2009; Lovell and Ford, 2012).

For some individuals the intake of FODMAPs may lead to an exacerbation of symptoms associated with IBS and other functional gut disorders (Gibson et al., 2015). The triggering of IBS symptoms by the consumption of FODMAPs is attributed to their slow or partial absorption in the small intestine. These carbohydrates are described as “fermentable” because they are likely to be fermented in the colon due to the absence, or reduced concentration, of hydrolase or because they are not absorbed completely (Staudacher et al., 2012). The gastro-intestinal tract has no hydrolase capable of degrading fructans or GOS (Kolida and Gibson, 2007; Teixeira et al., 2012; Fedewa and Rao, 2014; Staudacher et al., 2014). Polyols are only partially digested and absorbed in the small intestine and reach the colon, where they are fermented by bacteria (Murillo et al., 2016). In the small intestine, free fructose is absorbed thanks to GLUT5 and GLUT2 transporters. GLUT5 is a facultative transporter specific for fructose, and provides carrier-mediated facilitated diffusion. Although present along the whole length of the small intestine, GLUT5 shows low capacity leading to slow uptake of luminal fructose. GLUT2 is a low-affinity, facultative transporter for glucose, fructose, and galactose, that is activated in presence of high luminal glucose concentrations. Although fructose absorption is markedly enhanced in presence of luminal free glucose, if the latter is present in excess, the risk of fructose malabsorption is greater, because of the low affinity characterizing GLUT2. Other factors have been shown to alter fructose absorption, but the underlying mechanisms have not been totally elucidated (Gibson et al., 2007).

**FIGURE 1:**
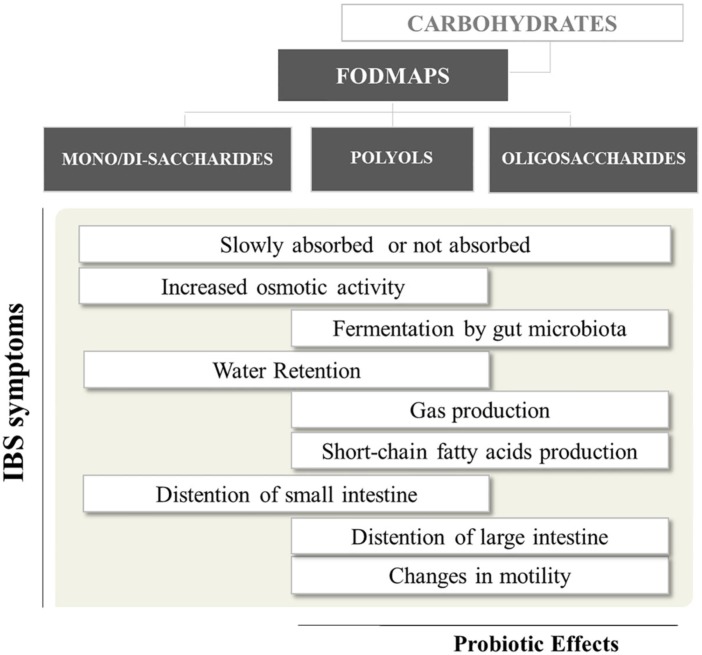
Short-chain carbohydrates and their relevance to gut health^1^. ^1^Bars more shifted to the left or right = effects attributed mostly to mono-disaccharides/polyols or oligosaccharides, respectively.

Delivery of FODMAPs to the lumen of the distal small intestine and proximal large intestine may have adverse consequences in IBS patients (**Figure 1**). FODMAPs are believed to induce symptoms by two main mechanisms: (i) drawing water into the small intestine, causing distension, swelling, and discomfort; (ii) the rapid fermentation of FODMAPs would generates gas, distending the colon and causing flatulence, swelling and discomfort. The first mechanism is mostly related to low molecular mass FODMAPs (e.g., fructose), which are osmotically active in the intestinal lumen. Therefore, volume of fluid entering the bowel increases, and this may provide a natural laxative effect in healthy people, but may contribute to diarrhea in IBS sufferers. Regarding the second mechanism, intestinal bacteria rapidly ferment FODMAPs (e.g., FOS) releasing short-chain fatty acids (SCFA) and gases (e.g., carbon dioxide, hydrogen, and methane) (Giorgio et al., 2015). Hence, FODMAPs malabsorption and their fermentation in the proximal colon can add gas and water to the luminal contents, leading to distension and the onset of other symptoms (Ong et al., 2010). Moreover, patients with visceral hypersensitivity respond exaggeratedly to gas and fluid distension caused by malabsorption of carbohydrates (Major et al., 2017).

Although all FODMAP have similar physiological effects, each FODMAPs subgroup has a different gastrointestinal response, depending on molecular mass, rapidity of absorption and osmolarity (Murray et al., 2014). Overall, slowly absorbed fructose and polyols have a greater osmotic effect per molecule than fructans. Conversely, oligosaccharides, being scarcely absorbed across the small intestine, will have greater fermentative effects than fructose and polyols (Giorgio et al., 2015).

A limited number of effective treatment strategies is available for IBS (Tuck et al., 2014). Modulation of FODMAPs’ intake in volume and type could be a means for controlling gut symptoms (Gibson et al., 2015); that is why it is extremely necessary to know the concentration of FODMAPs present in the greatest possible number of foods. Control of FODMAPs ingested as food components is known as “low FODMAPs diet” (Marsh et al., 2016). The recommended total daily intake of FODMAPs in patients with IBS ranges from 5 to 30 g per day (Staudacher et al., 2012; Böhn et al., 2015). The reduction of gastrointestinal and systemic symptoms by restriction of FODMAPs ingestion has been observed by many research groups (Roest et al., 2013; Pedersen et al., 2014; Böhn et al., 2015; Barrett, 2017). The dietary manipulation of FODMAPs ingestion was able to impact on the total amount of gastrointestinal gas production and the spectrum of gas produced (hydrogen vs. methane) in healthy individuals and in patients with IBS (Ong et al., 2010). Patients subjected to diets containing low amount of FODMAPs present milder symptoms of IBS, through dietary restriction and re-challenges tests, used to determine individual tolerance to various short-chain carbohydrates (Halmos et al., 2014; Tuck et al., 2014).

The adoption of a low-FODMAPs diet presents some difficulties related to the lack of clear “cut-off levels” for FODMAPs content in foods and no available information on FODMAPs content on food packages. The definition of cut-off values should consider the amount of each particular FODMAPs present in a food, the typical serving size of food consumed in a single sitting, and the commonly triggered symptoms in individuals with IBS. To build a comprehensive composition database for FODMAPs in food, based on “*ad hoc*” studies, is essential for defining cut-off values (Muir et al., 2009). In addition, in long term, the low FODMAPs diet could change the gastrointestinal microbiota (Halmos et al., 2014; Tuck et al., 2014). Changes in total bacteria abundance, relative abundance of bifidobacteria (Staudacher et al., 2012) and strongly butyrate producing clostridial groups or *Akkermansia muciniphila*, positively associated with health, were described (Halmos et al., 2014). Moreover, the low FODMAPs diet could reduce the intestinal production of SCFA (Ong et al., 2010).

## Beneficial Effects of Fodmap

Fermentable oligosaccharides, disaccharides, monosaccharides and polyols are fermented in the colon to SCFA, which exert multiple beneficial effects on human health. Apart from being a major energy source for colonocytes, SCFA play a major immunological role in the gut, and help various physiological functions including colonic mobility and blood flow, and gastrointestinal pH, which can influence uptake and absorption of electrolytes and nutrients (Den Besten et al., 2013; Tan et al., 2014). Furthermore, inulin-type fructans and GOS have been proposed for the treatment of metabolic endotoxaemia or low-grade inflammation in overweight/obese people (Morel et al., 2015; Fernandes et al., 2017). Some FODMAPs are being increasingly used in food industry as prebiotics, either as bioactive ingredients or as supplements, to promote colonic health. This kind of food/supplements should clearly be avoided by IBS patients. The higher FODMAPs’ intake compared with that of the low FODMAPs or habitual diets was associated with specific stimulation of bacterial groups with putative health benefits (Halmos et al., 2014).

## How Bread-Making May Change Fodmaps Level

The FODMAPs content of breads depends on the nature of the grain-ingredient, as well as on the processing parameters (Biesiekierski et al., 2011). Despite the scarcity of papers that have quantified FODMAPs in breads, different concentrations of fructans (0.1–1.7% of dry weight, dw) have been reported in rye, spelt, and wheat breads. Fructose (0.1–2.3% of dry weight), GOS (0.1–0.4% of dw), FOS (0.05–0.15% of dw), sorbitol and mannitol (in traces) were also found (Biesiekierski et al., 2011; Whelan et al., 2011; Laatikainen et al., 2016). Besides that, pseudo-cereals and legumes are used for enriching/fortifying bread with nutritionally important components (e.g., minerals, vitamins, and phenolic compounds). This would increase the dietary intake of FODMAPs (especially GOS), because pseudo-cereals and legumes contain remarkable amounts of raffinose and stachyose (Muir et al., 2009; Curiel et al., 2015).

As stated in the introduction, bread accounts for a major part of daily consumed FODMAPs. Although the individual concentration of each subgroup of carbohydrates that comprises the FODMAPs may be low – except fructans, which are found in relatively high concentrations – consider the total content of all the subgroups of FODMAPs is more important than the individual levels of each subgroup, since the main effects of its intake can be summative. The fact that bread is one of the most important carbohydrate sources of the daily diet makes its quantity of FODMAPs relevant to IBS-sufferers.

Baker’ yeast-leavened bread is typically obtained upon short fermentation (0.5–3 h), which causes a relatively limited hydrolysis of cereal components, including proteins and FODMAPs. On the opposite, sourdough biotechnology requires longer fermentation time. Sourdough is a complex microbial ecosystem, usually dominated by heterofermentative lactobacilli (e.g., *Lactobacillus brevis*, *Lactobacillus fermentum*, *Lactobacillus plantarum*, *Lactobacillus rossiae*, *Lactobacillus reuteri*, and *Lactobacillus sanfranciscensis*) and yeasts (e.g., *S. cerevisiae, Kazachstania exigua*, *Kazachstania humilis*, *Torulaspora delbrueckii, Wickerhamomyces anomalus*, and *Pichia kudriavzevii*). Microbial ecology of this ecosystem has been widely described in other reviews (De Vuyst et al., 2014; Gobbetti et al., 2016). Sourdough biotechnology may be exploited to improve flavor and texture, extend shelf-life and enhance nutritional and functional quality of leavened baked goods (Gobbetti et al., 2014). Most of these features are due to metabolic activities of LAB, especially on carbohydrates and proteins. During sourdough fermentation, the metabolism of carbohydrates depends on the available substrates, microbial and endogenous (flour) enzymes and interactions between microbial populations (e.g., competition and proto-cooperation). In detail, during typical (wheat and rye) sourdough fermentation, flour α-amylase hydrolyzes starch to maltodextrins, which are then converted by flour β-amylase into maltose, the most abundant fermentable carbohydrate in dough. At dough stage, microbial invertase rapidly cleaves flour sucrose into glucose and fructose (Gänzle, 2014). Glucose is used as energy source, whereas fructose may be reduced by heterofermentative LAB to mannitol. Through reduction of fructose to mannitol, these bacteria convert acetyl-phosphate to acetate (instead of ethanol), thus gaining an extra mole of ATP (Gobbetti et al., 2005). Overall, all the fermentable carbohydrates (sucrose, maltose, glucose, and fructose) are quickly depleted during the first hours of fermentation, whereas carbohydrates with a higher degree of polymerization (such as fructans) are used later. This leads to hypothesize that long fermentation, such as that typically relying on sourdough, can provide a more pronounced degradation of FODMAPs. Contrarily to starch and fermentable carbohydrates, the mechanisms behind FODMAPs degradation have received less attention.

Among FODMAPs, fructans in wheat could be degraded upon sourdough fermentation. Indeed, few sourdough LAB, such as *Lactobacillus amylovorus* and *Lactobacillus crispatus*, are able to metabolize fructans (Loponen and Gänzle, 2018). Muller and Lier (1994) described that fructans are converted to fructose and sucrose, which are further metabolized into lactic acid by homofermentative LAB or into lactic acid, acetic acid, ethanol and CO_2_ by heterofermentative LAB. In addition, reduction of fructose to mannitol, another FODMAP, frequently occurs in sourdough fermented by heterofermentative LAB (Gänzle, 2015). An extracellular, cell wall-associated β-fructosidase has been reported in strains of *Lactobacillus paracasei* as responsible for the degradation of fructans, leading to extracellular accumulation of sucrose and fructose (Makras et al., 2005; Goh et al., 2007).

*Lactobacillus plantarum*, *L. fermentum*, *L. brevis*, and *Lactobacillus buchneri*, isolated from vegetables, produce α-galactosidase, acting on GOS (Silvestroni et al., 2002). In addition, gene coding for levansucrase was expressed by *L. reuteri* LTH5448, isolated from sourdough, more than 100 fold in presence of raffinose (Teixeira et al., 2012). This strain, also possessing α-galactosidase activity, was used to ferment (37°C, 24 h) faba bean flour, which was added to gluten-free bread dough containing, among other ingredients, baker’s yeast and sorghum sourdough (previously fermented by the same strain). After 2 h of fermentation, GOS had been totally degraded. However, partial degradation of GOS to melibiose, manninotriose or mannino-tetraose was also observed when unfermented bean flour was used as ingredient, indicating that extracellular levansucrase rapidly acts on GOS, whereas subsequent intracellular α-galactosidase activity proceeds slower. Comparable partial degradation of GOS was also observed in the control gluten-free dough, wherein *L. reuteri* LTH5448 had not been used (Teixeira et al., 2012). This could be explained by the action of yeast’s invertase, which has a catalytic mechanism similar to levansucrase (Lammens et al., 2009). The invertase produced by *S. cerevisiae* is also able to degrade (partially) fructans to glucose and fructose. Indeed, during wheat whole meal bread-making, *S. cerevisiae* degraded almost 80% of the fructans initially present (Verspreet et al., 2013). Overall, yeasts may reduce the level of various FODMAPs during bread-making. For instance, Ziegler et al. (2016) showed that relatively long (4.5 h) baker’s yeast fermentation results quite effective for reducing up to 90% the dough levels of fructans, raffinose, and excess of fructose, in five *Triticum* species. Struyf et al. (2017) developed an efficient yeast-based strategy, using an inulinase-secreting *Kluyveromyces marxianus* strain to reduce by more than 90% fructans levels in bread, upon 2.5 h of dough fermentation.

Based on the above-mentioned enzymatic activities, some sourdough lactobacilli (*L. plantarum*, *L. fermentum*, and *L. brevis*) could ferment GOS, FOS, and fructans (Saulnier et al., 2007; Pan et al., 2009; Kunová et al., 2011). In turn, when lactobacilli hydrolyze fructans, polyols and excess fructose are generated. Strains of *L. plantarum* and *Lactobacillus curvatus* isolated from rye sourdough were able to ferment fructose, mannitol, and sorbitol (Bartkiene et al., 2017). Wheat germ subjected to long fermentation with autochthonous strains of *L. plantarum* and *L. rossiae* showed 87 and 45% decrease of fructose and raffinose, respectively, compared to the initial concentrations (Rizzello et al., 2010). Similarly, the combined use of *L. plantarum* and *L. brevis* to ferment (at 30°C, for 24 h) pulses flour resulted in a decreased concentration of raffinose (of up to ca. 64%), with respect to a control dough (obtained without bacterial inoculum) (Curiel et al., 2015).

Costabile et al. (2014) investigated the effects of breads, which differed in terms of fermentation time and biological leavening agent, on the colonic microbiota. *In vitro* batch culture experiments were run using feces from three subjects suffering from IBS and three healthy subjects. A significant increase in intestinal bifidobacterial populations, cultured *in vitro*, occurred after 8 h of fermentation with pre-digested sourdough breads. The same effect was not observed for breads produced with commercial yeast. Bifidobacteria do not produce gas and have been shown to benefit health of IBS patients (Clarke et al., 2012). δ-Proteobacteria and most Gemmatimonadetes species decreased in both IBS and healthy subjects upon 24 h-long exposure to sourdough bread. In addition, in IBS subjects the latter produced significantly lower cumulative gas after 15 h of exposure, compared to baker’s yeast breads. Overall this study suggests that sourdough bread may be beneficial for patients suffering from IBS, by influencing composition and metabolic profile of the human intestinal microbiota (Costabile et al., 2014). Another study has shown that low-FODMAPs rye breads, approximately 30% reduction of mannitol and fructans, caused less gastrointestinal symptoms, as abdominal pain, flatulence, stomach rum- and intestinal cramps (Laatikainen et al., 2016). A third study conducted by Pirkola et al. (2018) demonstrated that low-FODMAP rye bread leads to reduced colonic fermentation and flatulence severity when compared with regular rye bread consumption.

## Future Aspects

Future researches focusing on FODMAPs dietary contribution of bread will have to fill several knowledge gaps. First, information about FODMAPs content in food is limited, making difficult to understand their physiological importance (Muir et al., 2009; Giorgio et al., 2015). With special regard to bread, FODMAPs concentration depends on intrinsic characteristics (e.g., cereal variety, growing locations, climate, and season), ingredients of bread recipes and processing methods. This gap is hard to be filled also because different methodologies of extraction and detection may lead to different concentrations. When detected through chromatography, some FODMAPs co-elute, obliging to perform a second chromatographic step with two mobile phases. In addition, some FODMAPs are present at concentrations very close to the limit of chromatographic detection, which may cause errors in quantification (Paramithiotis et al., 2007). Finally, detection of some FODMAPs (e.g., fructans) requires a combination of chromatography and enzymatic hydrolysis (Muir et al., 2009; Biesiekierski et al., 2011). Overall the finding of well-standardized methods of FODMAPs detection would be a key-point for food researchers, industries and consumers. Besides knowing FODMAPs concentrations, it is of utmost importance to elucidate the degradation pathways of the different FODMAPs subgroups and how they affect intestinal microbiota. This would help to evaluate the different symptomatic responses of subjects and to guide researchers in designing foods targeted for any specific group of consumers (Tuck et al., 2014).

As discussed in the previous paragraph, LAB and yeasts may degrade FODMAPs. Compared to baker’s yeast, sourdough shows a great potential for driving FODMAPs concentrations of bread (and, overall, leavened baked goods), because it often includes both microbial populations. Therefore, future research should focus on how to combine enzyme activities of LAB and yeasts to reach the desired goal. In this context, given that some FODMAPs degrading enzymes are rather widespread in LAB, one pillar is the selection of microbial strains possessing such enzymes and their use as sourdough starters.

Finally, the effect of FODMAPs degradation on bread quality should be evaluated. For instance, sucrose and fructose, generated upon partial hydrolysis of fructans, may be further used by bacteria to synthesize exopolysaccharides. These compounds protect bacteria against various environmental stresses, improve texture and delay bread staling (Ketabi et al., 2008; Dertli et al., 2016). In addition some exopolysaccharides have prebiotic activity (Ketabi et al., 2008; Torrieri et al., 2014). On the other hand, eventually, the EPS produced can be a fructose-composed EPS and the degradation of this EPS during the dough preparation may again result in an increase in the fructose concentration, which is not favorable for IBS sufferers. Besides that, some LAB reduce fructose to mannitol, which would reduce the level of fructose but increase the other FODMAP. In this sense, the effects of sourdough fermentation on the content of FODMAPs will depend on the fermentation conditions and on which LAB species will be applied, offering a rather uncovered field to be clarified. As well as the possible increase in mannitol levels may affect the health of IBS-sufferers requires further studies.

Filling these knowledge gaps, it will be possible to modulate FODMAPs type and concentration in bread, allowing to reach two goals: (i) limiting the intake of FODMAPs in IBS patients to avoid undesirable gastrointestinal symptoms; (ii) attempting to increase their intake in healthy subjects.

## Author Contributions

LM, FM, and PF researched and wrote the article. MS, FM, MG, and JDDL critically revised the article.

## Conflict of Interest Statement

The authors declare that the research was conducted in the absence of any commercial or financial relationships that could be construed as a potential conflict of interest.
